# Nepalese migrants in Japan: What is holding them back in getting access to healthcare?

**DOI:** 10.1371/journal.pone.0203645

**Published:** 2018-09-07

**Authors:** Prakash Shakya, Masako Tanaka, Akira Shibanuma, Masamine Jimba

**Affiliations:** 1 Department of Community and Global Health, Graduate School of Medicine, The University of Tokyo, Tokyo, Japan; 2 Faculty of Global Studies, Sophia University, Tokyo, Japan; University of Sheffield, UNITED KINGDOM

## Abstract

**Introduction:**

Migrants are one of the most deprived and vulnerable groups who receive the least health services in the society. Only a few studies have been conducted on access to healthcare among migrants in Asia, despite hosting 75 million migrants. In Japan, Nepalese migrants constitute the largest South Asian community. Their number increased by three folds from 2011 to 2016. However, little is known about their access to health care in Japan. Based on Andersen's model, we examined the factors associated with access to healthcare among Nepalese migrants in Japan.

**Methods:**

We conducted a cross-sectional study among 642 Nepalese migrants residing in 10 prefectures of Japan. We used multivariable logistic regression model to explore the key predisposing, enabling, and need factors associated with access to healthcare among the migrants.

**Results:**

The migrants who had stayed in Japan longer were more likely to perceive better access to a doctor/health worker (AOR = 1.11, 95% CI 1.03–1.19).The migrants were more likely to perceive better access to a doctor/health worker (AOR = 1.79, 95% CI 1.17–2.73) when they did not need Japanese language interpreter during visit to health facilities. They were also less likely not to see a doctor/health worker when needed (AOR = 0.34, 95% CI 0.21–0.56). The migrants were less likely to perceive better access to a doctor/health worker when they had not paid the health insurance premium regularly (AOR = 0.21, 95% CI 0.13–0.33).Their low perception of better access to a doctor/health worker was also associated with self-rated health status as poor or fair (AOR = 0.60, 95% CI 0.41–0.89).

**Conclusion:**

Nepalese migrants have poor access to health care in Japan. The key factors associated with their access to health care are the length of stay (predisposing factor), Japanese language skill and health insurance (enabling factors) and self-rated health status (need factor).

## Introduction

Migrants are one of the most deprived and vulnerable groups in society[1]. Their vulnerability is related to several factors including process of migration, low socio-economic status, ethnicity, communication, and immigration policy[2]. These factors also play crucial role in shaping their overall health status in the host countries. Poor health causes additional difficulties for migrants to adapt to their host societies[2]. It also violates the notions of "health and human rights" and "equity in health"[3, 4].

Migrants’ access to health care is an important area of concern in global health. Studies have identified “healthy migrant effect”, where migrants are healthier upon arrival compared to the general population in the host country [5, 6]. However, such effect decreases over time as migrants face several challenges in the host environment [2, 7]. Moreover, compared to the host population, migrants have less access to health care which contributes to their poor health status[8]. Access to health care is generally defined as the timely use of personal health services to achieve the best possible health outcomes[9]. Andersen's model provides a whole system approach to analyze both potential and realized access to health care among migrants[2]. It divides the individual and contextual determinants of access to health care into three major components; predisposing, enabling, and need factors[10]. Predisposing factors include demographic and social characteristics such as age, sex, ethnicity, education, occupation, and social ties. Enabling factors include financing and organizational factors which enable health service utilization such as health insurance, employment status and language proficiency. Need factors include perceived need for health services such as self-rated general and mental health status, and evaluated need such as clinicalevaluation at a health facility.

A limited number of studies have been conducted on access to health care among migrant populations in Asia, despite hosting 75 million migrants. Of 450 Central Asian migrants in Kazakhstan, roughly half had lower utilization of health services and poor self-reported health status[7]. Only one third (out of 408)Nepalese migrants in Qatar, Saudi Arabia and United Arab Emirates had health service insurance provided by their employers which led to their poor access to health care [11]. The socio-cultural environment of Asia is different from that of the western world, which may reveal different dynamics of health care access among its migrants.

Evidence is scarce on migrants' access to health care in Japan. Japanese immigration policy favors migrant control over migrant rights, which has negative consequences on their access to health care[12, 13]. Migrant health outcomes are worse in comparison with the general population because of untimely access to health care. For example, the incidence of tuberculosis is higher among migrants than the Japanese population due to inadequate access to diagnosis and treatment[14].

Japan is a popular destination for Nepalese migrants. About 60,000 Nepalese were residing in Japan in 2016 and it is the largest South Asian community in the country[15].From 2011 to 2016, there has been a threefold increase in the number of Nepalese in Japan[15]. About 20,000 of them are students, most of whom are studying in Japanese language schools and professional training colleges while a smaller number attend universities[15]. Most of the students are also engaged in part-time jobs in convenience stores, restaurants, delivery services and housekeeping jobs. A majority of the Nepalese male migrants are also working as cooks in Indo-Nepali restaurants under legal status as skilled labors. Another large group is the wives of those cooks, who are engaged in part-time jobs under the legal status of dependents. A small number of the Nepalese migrants have office jobs in companies or have their own business.

Most of these Nepalese students, cooks and their dependents may be in vulnerable health conditions. However, little is known about their access to health care in Japan. This study examined the factors associated with access to health care among Nepalese migrants in Japan.

## Methods

### Study design and area

We conducted this cross-sectional study in the following 10 prefectures of Japan: Tokyo, Fukuoka, Osaka, Gunma, Tochigi, Hyogo, Kanagawa, Chiba, Aichi, and Saitama. Most of the Nepalese migrants are residing in these prefectures. Among them, Tokyo had the largest Nepalese population of 18,869 followed by Fukuoka (4,876) and others in 2015[15].

### Sampling strategy and participants

We recruited the migrants based on convenience sampling method, in each study prefecture. We visited language schools, professional training colleges, Indo-Nepali restaurants, and other work stations in the study area to recruit the migrants. We first identified key informants, who were the leaders of local Nepalese communities in each study area. The key informants helped us identify the language schools, professional training colleges, Indo-Nepali restaurants and other work stations in the study area where most of the Nepalese migrants are studying and working. They also introduced us to other Nepalese migrants residing in respective prefecture.

We recruited migrants (both male and female) who fulfilled the following eligibility criteria.

#### Inclusion criteria

- Nepalese citizen
- Aged 18–60 years
- Who have stayed continuously or with interruption for at least three months in Japan
- Willing to participate in the study voluntarily

#### Exclusion criteria

- Those who cannot read and write in Nepali language

### Dependent variables

#### Access to health care

We measured access to health care by two binary variables. We asked the migrants (a) if they currently have proper access to a doctor/health worker and (b) if in the past year, they needed to see a doctor/health worker for an illness but did not. Both items have been previously used to examine access to health care among migrants in Central Asia[7]. The first variable measures the perceived potential access to health care and the second variable measures the unmet utilization or realized access to health care.

### Independent variables

We chose the following independent variables based on the three major components of individual and contextual determinants of Andersen's model of access to health care; predisposing, enabling and need factors[16].

#### Predisposing factors

We asked questions on socio-demographic characteristics such as sex, age, marital status, ethnicity, and educational status. We also asked them questions on their visa/legal status and length of stay in Japan.

#### Enabling factors

We assessed the enabling factors for the migrants' access to health care by asking questions about their employment status, health insurance premium payment and their need of a Japanese language interpreter. We also measured their perceived social support using Multi-dimensional Scale of Perceived Social Support (MSPSS)[17]. It is a 12-item scale and items are scored from one (very strongly disagree) to seven (very strongly agree). The possible scores range from 12 to 84. This scale has already been tested for its validity among Nepalese migrants[18]. Its Cronbach's alpha was 0.97 for this study.

#### Need factors

We measured the need factors by assessing the migrants' overall and mental health status. We asked them to rate their overall health status on a 5-point Likert scale (excellent, very good, good, fair, or poor). This single-item instrument has been used widely in previous studies and also among migrants in Central Asia[7].

We assessed the mental health status using measures of stress, depressive symptoms and anxiety. We measured stress in the past month using the Perceived Stress Scale (PSS). It is a 10-item scale with scores ranging from zero (never) to four (very often). The possible scores range from zero to 40. This scale has already been tested for its validity and used among the Nepalese population[19]. Its Cronbach's alpha was 0.85 for this study.

We measured depressive symptoms using a Center for Epidemiologic Studies Depression (CESD) scale. It is a 20-item scale with scores ranging from zero (never or rarely or none of the time or less than 1 day) to three (mostly or allof the time [5–7] days). The possible scores range from zero to 60. A score of 16 or more indicates depressive symptoms. This scale has already been tested for its validity and used among the Nepalese population[19]. Its Cronbach's alpha was 0.87 for this study.

We measured anxiety in the past month using Symptoms Checklist-90-R (SCL-90-R) Anxiety Subscale. It is a 10-item scale with scores ranging from zero (not at all) to four (extremely). The possible scores range from zero to 40. This scale has already been tested for its validity and used among the Nepalese population[19].Its Cronbach's alpha was 0.94 for this study.

### Data collection

We collected the data using self-administered questionnaire in Nepali. We pretested it among 40 migrants and rephrased a few questions based on the pretest results. We collected the data from April to July, 2016. The first author and eight research assistants collected the data. We visited language schools, professional training colleges, Indo-Nepali restaurants, and other job sites in the study area to recruit the migrants. We distributed questionnaires to a total of 981 potential participants who fitthe eligibility criteria of this study. We obtained a total of 765 filled questionnaires back. Among them, we used 642 questionnaires as we had to exclude 26 questionnaires due to the lack of written consent and 97 questionnaires due to missing data.

### Data analysis

We included a total 642 migrants in the data analysis. We analyzed the general characteristics, migration characteristics and access to health care among the migrants, descriptively. We conducted multivariable analysis using logistic regression model to examine the factors associated with access to health care among Nepalese migrants. We included the following independent variables in the regression model, based on Andersen’s model of access to health care: a) Predisposing factors: age, sex, marital status, ethnicity, education obtained in Nepal before migration, length of stay in Japan, and visa/legal status b) Enabling factors: employment status in Japan, need of Japanese language interpreter during visit to health facilities, payment of the health insurance, and perceived social support score: c) Need factors: self-rated health status, stress score, depressive symptoms score, and anxiety score. We categorized the ethnicity into three groups;Brahmin/Chhetri/Thakuri, Janajati, and Dalit. Brahmin, Chhetri and Thakuri are regarded as relatively well-off ethnicities. Dalit is considered an underprivileged ethnicity and Janajati is an indigenous ethnicity. In the multivariable regression model, we included the categorical variables after transforming them into multiple dichotomous variables (dummy encoding). We treated the Likert scale variables as ordinal variables and included them in the model after transforming into multiple dichotomous variables. We did not find any multicollinearity among the predictor variables. We set the statistical significance at *p*<0.05. We conducted all the analyses using STATA software, version 13.

### Ethics statement

This study was reviewed and approved by the Research Ethics Committee of the Graduate School of Medicine of the University of Tokyo (Serial number 11102). We obtained written informed consent from the migrants. They participated voluntarily. We did not ask for or record any migrant's name, only an identification code was used.

## Results

### General characteristics

Table 1 summarizes the general characteristics of the migrants (N = 642). They had a mean age of 28.3 years (SD 7.0, Median 26.0, Range 18.0–58.0). About 73% of them were men. About 47% of them were married. About 17% of them had obtained less than higher secondary education in Nepal, 50.0% had higher secondary level, and 33.3% had bachelors degree or above. In Nepal, higher secondary level is achieved after completing 12 years of education, also known as 10+2 standard. In Nepal, before coming to Japan, 49.2% of them were students, 20.3% were working in service sector, and 11.1% had their own business.

**Table 1:** General characteristics of Nepalese migrants in Japan.

Variables	N = 642	Mean	%	SD	
**Age (years)**		28.3		7.0	
**Sex**					
Male	467		72.7		
Female	175		27.3		
**Ethnicity**					
Brahmin/Chhetri/Thakuri	359		56.0		
Janajati	237		37.0		
Dalit	46		7.2		
**Marital status**					
Married	302		47.0		
Unmarried	340		53.1		
**Education obtained in Nepal before migration**					
Less than higher secondary	107		16.7		
Higher secondary	321		50.0		
Bachelors degree or above	214		33.3		
**Occupation in Nepal before migration**					
Business	71		11.1		
Agriculture	22		3.4		
Service	130		20.3		
Household (unpaid)	42		6.6		
Student	316		49.2		
Unemployed	36		5.6		
Others	25		3.9		

### Migration characteristics and perceived social support

Table 2 shows the migration characteristics and perceived social support of the migrants measured by MSPSS (N = 642). Their mean length of stay in Japan was 3.9 years (SD 3.6, Median 3.0, Range 0.2–25.0). About 57% of them were on student visa, 9.8% were skilled labor (cook visa), and 14.8% were dependent. About 32% of them were students in professional training colleges and part-time employees, 19.6% were students in language schools and part-time employees,18.6% were full-time employees, and 12.3% were part-time employees. All the migrants had a mean MSPSS score of 56.8 (SD 23.7, Median 64.0, Range 12.0–84.0).

**Table 2:** Migration characteristics and perceived social support among Nepalese migrants in Japan.

Variables	N = 642	Mean	%	SD
**Length of stay in Japan (years)**		3.9		3.6
**Visa/legal status**				
Skilled labor (Cook visa)	63		9.8	
Student	363		56.6	
Dependent	95		14.8	
Engineer / Specialist in Humanities / International Services	23		3.6	
Permanent resident/Spouse or child of permanent resident	20		3.1	
Designated activities (refugee applicant)	46		7.2	
Business manager	14		2.2	
Others	18		2.8	
**Employment status in Japan**				
Engaged in own business (self employed)	41		6.4	
Full-time employee	119		18.6	
Part-time employee	79		12.3	
Student (language school) and part-time employee	126		19.6	
Student (professional training college) and part-time employee	202		31.5	
Student (university) and part-time employee	39		6.1	
No job	36		5.6	
**Perceived social support score**		56.8		23.7

### Self-rated health status, health insurance and mental health outcomes

Table 3 presents the self-rated health status, health insurance and mental health outcomes of the migrants in Japan (N = 642). About 51% of them rated their health status as poor or fair. About 35% of them had not paid the health insurance premium for a year or more. About 82% of them said that the health insurance is beneficial. About 62% of them said that the cost of health insurance is expensive. All the migrants had a mean stress score of 17.6 (SD 6.2, Median 18.0, Range 0.0–37.0) and a mean anxiety score of 4.4 (SD 6.7, Median 1.0, Range 0.0–40.0). About 24% of them had depressive symptoms (score ≥ 16).

**Table 3:** Self-rated health status, health insurance and mental health outcomes among Nepalese migrants in Japan.

Variables	N = 642	Mean	%	SD
**Self-rated health status**				
Good/very good/excellent	314		48.9	
Poor/fair	328		51.1	
**Payment of the health insurance premium**				
Regular	416		64.8	
Irregular (Not paid for a year or more)	226		35.2	
**Do you think health insurance is beneficial to you?**				
Yes	526		81.9	
No	66		10.3	
Don't know	50		7.8	
**Do you think the cost of health insurance is expensive to you?**				
Yes	396		61.7	
No	174		27.1	
Don't know	72		11.2	
**Stress score**		17.6		6.2
**Depressive symptoms score**				
Yes (score ≥ 16)	156		24.3	
No (score <16)	486		75.7	
**Anxiety score**		4.4		6.7

### Access to health care and need of language interpreter during visit to health facilities

Table 4 shows the access to health care and need of language interpreter during the visit to the health facilities for the migrants in Japan (N = 642). About 69% of them did not perceive that they had proper access to doctor/health worker in Japan. About 32% of them needed to see doctor/health worker in the past year, but did not. About 70% of them said that they needed Japanese language interpreter during their visit to the health facilities.

**Table 4:** Access to health care and need of language interpreter during visit to health facilities among Nepalese migrants in Japan.

Variables	N = 642	%
**Perceived better access to a doctor/health worker**		
Yes	198	30.8
No	444	69.2
**In the past year, needed to see a doctor/health worker, but did not**		
Yes	203	31.6
No	439	68.4
**Need of Japanese language interpreter during visit to health facilities**		
Yes	449	69.9
No	193	30.1

### Factors associated with access to health care among migrants in Japan

Table 5 presents the factors associated with access to health care among Nepalese migrants in Japan (N = 642). The following predisposing, enabling, and need factors were either directly or inversely, significantly associated with access to health care in multivariable regression model.

**Table 5:** Factors associated with access to health care among Nepalese migrants in Japan.

	Perceived better access to a doctor/health worker (N = 642)			Needed to see a doctor/health worker, but did not (N = 642)		
Variables	AOR	95% CI	p value	AOR	95% CI	p value
***Predisposing factors***						
**Age**	1.02	0.97–1.06	0.437	0.99	0.95–1.03	0.488
**Sex**						
Male	Ref.					
Female	1.00	0.64–1.66	0.999	0.81	0.50–1.29	0.469
**Marital status**						
Unmarried	Ref.					
Married	1.14	0.69–1.90		0.97	0.57–1.66	0.927
**Ethnicity**						
Brahmin/Chhetri/Thakuri	Ref.					
Janajati	0.75	0.49–1.33	0.165	1.04	0.68–1.60	0.811
Dalit	0.66	0.31–1.41	0.262	2.28	1.14–4.59	0.015
**Education obtained in Nepal before migration**						
Less than higher secondary	Ref.					
Higher secondary	1.83	0.92–3.62	0.099	0.87	0.47–1.59	0.723
Bachelors degree or above	1.81	0.92–3.57	0.097	0.64	0.34–1.19	0.177
**Length of stay in Japan (years)**	1.11	**1.03–1.19**	**0.008**	0.94	0.87–1.02	0.198
**Visa/legal status**						
Student	Ref.					
Skilled labor (cook) or dependent	1.02	0.34–3.06	0.879	0.84	0.29–2.46	0.895
Designated activities (refugee applicant)	1.07	0.28–4.06	0.937	0.62	0.18–2.15	0.576
Others	0.78	0.23–2.60	0.530	0.44	0.13–1.57	0.294
***Enabling factors***						
**Employment status in Japan**						
Full-time employee	Ref.					
Part-time employee	1.09	0.49–2.42	0.800	1.02	0.50–2.11	0.971
Student and part-time employee	1.37	0.43–4.37	0.708	0.38	0.12–1.21	0.129
Others	0.96	0.46–2.00	0.934	1.05	0.50–2.20	0.986
**Need of Japanese language interpreter during visit to health facilities**						
Yes	Ref.					
No	1.79	**1.17–2.73**	**0.015**	0.34	**0.21–0.56**	**<0.001**
**Payment of the health insurance premium**						
Regular	Ref.					
Irregular (Not paid for a year or more)	0.21	**0.13–0.33**	**<0.001**	4.09	**2.75–6.08**	**<0.001**
**Perceived social support score**	1.01	1.00–0.99	0.238	1.00	0.99–1.01	0.775
***Need factors***						
**Self-rated health status**						
Good/very good/excellent	Ref.					
Poor/fair	0.60	**0.41–0.89**	**0.013**	1.03	0.70–1.53	0.945
**Stress score**	1.01	0.96–1.01	0.953	0.99	0.96–1.03	0.536
**Depressive symptoms score**	1.02	0.99–1.05	0.058	1.00	0.98–1.04	0.833
**Anxiety score**	0.98	0.94–1.01	0.234	1.01	0.98–1.04	0.639

#### Predisposing factors

Migrants who had stayed in Japan longer were more likely to perceive better access to a doctor/health worker (AOR = 1.11, 95% CI 1.03–1.19).

#### Enabling factors

The migrants were more likely to perceive better access to a doctor/health worker (AOR = 1.79, 95% CI 1.17–2.73) when they did not need Japanese language interpreter during visit to health facilities compared to those who needed. They were also less likely not to see a doctor/health worker when needed (AOR = 0.34, 95% CI 0.21–0.56). The migrants were less likely to perceive better access to a doctor/health worker (AOR = 0.21, 95% CI 0.13–0.33) when they had not paid the health insurance premium for a year or more compared to those who paid regularly. They were also at a higher risk of not seeing a doctor/health worker when needed (AOR = 4.09, 95% CI 2.75–6.08).

#### Need factors

The migrants were less likely to perceive better access to a doctor/health worker (AOR = 0.60, 95% CI 0.41–0.89) when they rated their health status as poor or fair compared to those who self rated as good or very good or excellent.

## Discussion

Nepalese migrants had poor access to health care in Japan. More than half of them did not perceive better access to a doctor or health worker (potential access) and about one third of them needed to see a doctor or health worker in the past year, but did not (realized access).The following key factors were associated with their poor access to health care. The key predisposing factor was shorter length of stay. The key enabling factors were need of Japanese language interpreter and not paying the health insurance premium regularly. The key need factor was poor or fair self-rated health status.

Access to health care includes both potential and realized access[2]. Perceived access to health care measures potential access. Realized access means the actual use of health services. It is measured by utilization of health services when needed. Indicators of potential and realized access are interlinked with each other. In our study, both potential and realized accesses were poor among Nepalese migrants in Japan. Comparable results were found among labor migrants in other regions of Asia. In Kazakhstan, about 45% of Central Asian migrants needed to see doctor in the past year but did not[7]. In Lebanon, only10% of Indian migrants had visited clinic/hospital at least once in last three years[20].

Several factors contributed to poor access to health care among the migrants. One of them was the lack of Japanese language skill. More than two-third of the migrants felt the need of a Japanese language interpreter during the visit to the health facilities in Japan. They were also less likely to have better access to health care. Differences in language between health care professionals and patients act as a barrier to health care access[21]. Its consequences range from miscommunication to inefficient use of health services[22–24]. The language barrier causes difficulty for health workers when seeking the patients' vital medical history, while the patients may have problems understanding and following through the prescribed treatment[25]. Language difficulty also decreases the confidence of the patients and they may feel embarrassed to seek out the services[25]. Overall, it results in low utilization of health services. Similar results were found among Latinos with limited English proficiency in a study conducted in United States[26]. Availability of professional interpreter services can improve the access to health care among migrants with poor local language skills[27]. This will reduce miscommunication between health workers and patients, and encourage migrants to utilize health services.

In this study, about 35% of the migrants had not paid health insurance premiums for a year or more. They were also less likely to have better access to health care in Japan. Being insured significantly increases health service utilization and decreases the delay of health care[28–30]. In Japan, it is mandatory for all citizens and foreigners residing for a year or more, to enroll in one of the public health insurance schemes[31]. Nepalese migrants get enrolled in National Health Insurance (NHI) scheme when they first enter Japan. However, many do not pay the insurance premium regularly afterwards. Failure to pay for many years results in heavy cost of back payments of premiums for the period they had not paid for. Thus, those migrants become reluctant to utilize health services in Japan. This finding is comparable with a study among Latin American migrants in Japan[32].

The reasons for not paying the premium may be due to the perceptions of the migrants on costs and benefits of the health insurance scheme. More than half of them perceived the health insurance cost to be expensive. About 18% of them thought either the health insurance is not beneficial to them or they did not know about it. These perceptions may be due to the lack of knowledge and awareness about health insurance benefits. In Nepal, National Health Insurance Policy was created in 2013[33]. The Government of Nepal formed the legal framework for a social health security scheme recently in 2015, which is yet to be implemented[33]. Out-of-pocket payment remains the major way of financing health care in Nepal[34]. So, the majority of the migrants were never exposed to health insurance culture before, which may explain their lack of awareness regarding health insurance costs and benefits.

The migrants were more likely to have better access to health care when they had stayed for a longer period in Japan. Newcomer migrants may have less knowledge about the availability and accessibility of the health services in Japan. They may also have lesser proficiency in Japanese language compared to those who have stayed for longer period. Moreover, they may have low level of acculturation in the host country, which can restrict them from health care access[35, 36]. Comparable results were found in previous studies. In United States, newly arrived immigrants had access to health care at similar levels as those without health insurance[37]. In Netherlands, Surinamese migrants were more familiar with local health services and had more access, if they had stayed for a longer period[38].

We did not find a significant association between self-rated health status and realized access or unmet utilization of health care. Those who perceive their health status as poor tend to utilize the health services frequently [30, 39]. However, in this study the migrants were less likely to perceive better access to health care when they self-rated their health status as poor or fair compared to those who self-rated as good, very good, or excellent. There may be also the possibility of reverse association. Those who did not perceive better access to health care might rate their health status as poor or fair because they did not have opportunity to utilize the health care services in Japan when they felt sick.

The findings of this study should be interpreted in line with the following limitations. Convenience sampling might have introduced selection bias and the sample may not be representative of the Nepalese population in Japan. Such selection bias may have over or underestimated the association of the factors with access to health care. Migrants are a hard to reach population, so it may be unfeasible to get a representative sample. Methods such as convenience sampling provide the best opportunity to recruit a relevant sample [40]. We attempted to minimize such bias by collecting data in 10 different prefectures of Japan, where most Nepalese migrants are residing. Moreover, the included migrants had various visa statuses and the response rate was also fairly high. We collected the data based on self-reporting from the migrants rather than documentation from services attendances. This might have affected the results due to possible recall bias and social desirability bias.

We did not explore the system level and provider level factors which may also influence the access to health care among Nepalese migrants in Japan[25]. The system level factors include system characteristics such as immigration policy, structural stigma and organizational barriers. The provider level factors include provider characteristics such as communication skills and cultural knowledge. Andersen’s model of access to health care is a widely used model. However, one of the major drawbacks of this model is the lack of factors associated with provider level services and policy. Future studies should also explore these factors which are of paramount importance for migrants’ access to health care.

Another important limitation is exclusion of 123 participants from the recruited sample. Although we did not find any Nepalese migrant who met the exclusion criteria of not being able to read and write in Nepali language, we excluded 26 migrants due to lack of written consent and 97 migrants due to missing data. However, we did not find any systematic pattern among the migrants who did not provide written consent and those with missing data. We excluded the missing data from the analysis by listwise or case deletion. It might have following implications in the study. First, the absence of data might have reduced the statistical power. However, data collection was maximized anticipating the missing data. Second, the lost data might have caused bias by over or underestimating the results. Multiple imputation of the missing data might have reflected the uncertainty associated with the estimation[41]. Third, the lost data might have reduced the representativeness of the sample.

Despite these limitations, this study is important as it first explored the factors associated with access to health care based on Andersen's model, among migrants in Asia. This is also the first study conducted on health issues of Nepalese migrants in Japan.

## Conclusion

Nepalese migrants have poor access to healthcare in Japan. The key factors associated with their better access to healthcare are the longer length of stay (predisposing factor), not needing the Japanese language interpreter during the visit to health facilities and paying the health insurance premium regularly (enabling factors), and self-rated health status as good or very good or excellent (need factor). Interventions should focus on reducing the language barrier between migrants and health workers, possibly by providing the professional interpreter service in health facilities. Proper measures should be taken to encourage the migrants to pay the health insurance premium regularly, particularly the newcomer migrants. Future studies should also examine the influence of system level and provider level factors on access to healthcare among migrants in Japan.
